# Current state, equality level and trends of self-rated health among old adults with intact physical condition

**DOI:** 10.1186/s12889-023-15970-8

**Published:** 2023-06-02

**Authors:** Weicun Ren, Clifford Silver Tarimo, Zhang Liang

**Affiliations:** 1 School of Political Science and Public Administration, Wuhan University, Wuhan, China; 2 Wuhan University Health Governance Research Centre, Wuhan University, Wuhan, China; 3 College of Public Health, Zhengzhou University, Zhengzhou, China; 4 Department of Science and Laboratory Technology, Dares Salaam Institute of Technology, Dar Es Salaam, Tanzania

**Keywords:** Old adults, Self-rated health, Intact physical condition, Equality, VAR

## Abstract

**Background:**

Self-rated health among old adults (SHOA) indicates individuals' subjective assessments and evaluations of their overall health based on objective physical circumstances. The purpose of this study was to analyze the current state and influencing factors of the subjective perception-based self-rated health (SH) by qualifying selected older adults with similar objective physical conditions, as well as to explore the equality and changing trends of SHOA based on influencing factors.

**Methods:**

This study designed a cross-sectional study, conducted in three provinces in east, central and west China, and included 1,153 older adults (> = 60 years) with intact physical condition (IPC). The current state of SHOA and its influencing factors were analyzed using mean comparisons and Logistic regression (LR) models. The equality level and trend of SHOA's effect on health literacy, health habits, and access to health care were determined using the Lorenz curve, Gini coefficient, and Vector Autoregression (VAR) model.

**Results:**

The mean SHOA with IPC was 74.37 ± 13.22. Findings from LR modeling indicated that SHOA with IPC was mainly influenced by age and communication methods (*P* < 0.05). It was also observed that the total Gini coefficient of the allocation of SHOA with IPC based on communication methods was equal to 0.0188, and the VAR results showed that the total effect of change in SHOA on health literacy among older adults was negative and its duration of the effect exceeded 50.

**Conclusions:**

The SHOA with IPC was shown to be better and was primarily influenced by age and communication methods. The observed effect of SHOA on health literacy was negative and lasting. To improve SHOA with IPC even further, policymakers could consider promoting the use of modern and convenient communication methods (such as smartphones) through training and purchasing subsidies, as well as focusing on increasing sustained attention and promoting health literacy and behavior among older adults with improved SH.

**Supplementary Information:**

The online version contains supplementary material available at 10.1186/s12889-023-15970-8.

## Introduction

Self-rated health (SH) is an assessment of people health's overall condition, reflecting their subjective perceptions and expectations of their general health based on their objective physical condition, and plays an important role in the enhancement of personal well-being and health resource utilization [1–3]. Studies have shown that SH is a strong predictor of morbidity and mortality [4–6]. An RJ et al. [7] found that improving the self-rated health of older adults (SHOA) was effective in preventing the onset of depression. Objective perceptions of physical condition are mainly related to pain and mental status [8]. The effect of physical state on people's daily life and mobility was also shown to have a direct effect on their quality of life [9]. Due to subjective perceptions, individuals with normal physical conditions and no abnormal experiences such as pain do not have the same SH [10]. Because of their age, older persons are more anxious about the likelihood of their physical circumstances to change, resulting in variations in SH when their objective physical situations are similar [11]. In the eastern, central and western Chinese provinces of Guangdong, Hubei and Guizhou, the average life expectancy of residents had exceeded 70 years by 2020, with a total of 23.69 million people aged 65 and above, accounting for 10.66% of the total population [12]. How to improve the SHOA has become a real problem that families, government and society have to face.

The distinctions between the subjective perception-based assessment of older individuals' health status and the factors influencing it, while the fundamental physical circumstances remain intact, are not yet well understood. Previous studies have shown SHOA to be associated with various personal characteristics such as age, sex, pre-retirement occupation, education level, and communication methods [13, 14]. A study conducted by Andersen FK et al. [15] in Denmark found that SH declined with age in older adults, but the trends in comparative SH with age were not identical in cross-sectional and longitudinal analyses. Meanwhile, Jung MS [16] concluded that sex differences are important in reporting SH, particularly the differential effects of subjective and objective executive functions on SH across sex groups. A study conducted in Korea also found that women's SH was related to marital status and family type [17]. Existing studies have revealed a strong association between pre-retirement occupation and SH, for example, a study based on data from the Chinese Labor Force Dynamics Survey showed that being a state employee had a significant positive effect on SHOA [18]. The results showed that those with higher education showed a slight improvement in SH, while those without higher education did not [19]. Existing research also suggests that adequate health insurance contributes to improved SHOA [20] and that the use of communication methods such as the internet mediates the socioeconomic status-SH relationship [21].

In addition, SHOA is closely related to its own health characteristics such as health literacy, health behaviors and health services accessible [22, 23]. It is often believed that having more health knowledge is beneficial to health, and it is only when there is awareness in the mind that changes can be made in real life [24]. Qiu F et al. found that knowledge of diet was a protective factor for SH [25]. Existing studies indicate that daily exercise is advantageous to SHOA, although there is less consensus about the impact of smoking and drinking status on SHOA [26, 27]. Positive assessments of SH, on the other hand, were discovered to have a protective effect on health behaviors, such as higher levels of exercise and lower levels of smoking and drinking [28]. Based on the fact that satisfaction with health services is associated with positive self-perceptions of health [29], Ferreira AM et al. [30] argued that in smaller cities, people have higher accessibility to health services and increased positive self-perceptions of health due to a closer relationship between health workers and users.

In summary, there are numerous studies on SHOA. However, there are relatively few studies on SHOA based on matched physical conditions, and the influencing factors while specific pathways that affect SHOA with intact physical condition (IPC) remain unclear. Hence the current study has statistically evaluated SHOA with IPC and investigated the elements that influence SHOA, as well as the magnitude and trends of those aspects.

To help develop effective strategies for the provision of old adult services and to improve the utilization of personal health resources and well-being of older adults, this study analyzed the current state and influencing factors of SHOA with IPC using mean comparison and Logistic regression (LR) model. The equality level of SHOA with IPC and the trend of the effect on health literacy, health behaviors, and health services accessible were determined by Lorenz curve and Gini coefficient, Vector Autoregression (VAR) model.

## Methods

### Data source

This current study was based on the original data from the "Chinese residents' health service needs survey in the New Era" conducted in July–August 2018. The survey covered a wide range of information, including basic information on the number of household members and socioeconomic status, individual demographic characteristics, SH, demand and utilization of preventive health services, and demand and utilization of health services, using a questionnaire with good reliability and validity [31, 32]. A three-stage, stratified, random sampling technique was used to select respondent households from three provinces in east, central and west China, respectively. Further details on sampling design can be accessed elsewhere [31, 33]. The survey was conducted by a group of undergraduate and graduate students with relevant expertise and training using a face-to-face question-and-answer format, and questionnaires were completed on site. All participants were informed of the details of the study before participating and the informed consent was obtained from all participants and their legal guardians. The questionnaires were collected to exclude the presence of missing values and logical errors in the survey variables. The content and procedures of the study were approved by the Ethics Committee of Tongji Medical College, Huazhong University of Science and Technology to be in compliance with international and national ethical requirements for biomedical research, and was performed in accordance with relevant guidelines and regulations (IORG number: IORG0003571).

This study employed a sample consisting of individuals aged 60 or above with IPC. Considering the absence of missing responses for key variables such as age and SH, a total of 1,153 older adults were finally included in the analysis.

### Definitions of dependent variables

In this study, the SHOA who perceived themselves as having IPC were used as the dependent variable. Both IPC and SH of older adults were defined based on the results of the EQ-5D-3L scale [34]. In this context, SHOA is defined as the SH score (0–100 points) on the scale. The closer SH score is to 100, the better SHOA and vice versa. The physical conditions of older adults were assessed by their perceptions and evaluations of their mobility, self-care (toileting, dressing, toileting, etc.), ability to perform daily activities (work, reading, or housework), physical pain, and anxiety or depression. For the purpose of this study, an older adult with IPC was defined as having no difficulties with mobility, self-care, or ability to perform daily activities, and not feeling physical pain, anxiety or depression.

### Definitions of independent variables

Referring to existing studies in the relevant literature, many variables were included in the regression models as potential determinants of SHOA and its inequalities [13, 14, 22, 23]. The independent variables were divided into four parts: (1) The basic personal profile of old adults was reflected by eight indicators including age, sex, residence, marital status, education level, pre-retirement occupation, medical insurance, and communication methods [31]; (2) Health literacy included ability to communicate fluently with medical staff, proactive reading of information related to their health, changing their lifestyle according to the guidance of health information; (3) Health behaviors of older adults obtained from smoking status, drinking status, and number of physical exercise. Among them, smoking was defined as continuous or cumulative smoking for 6 months or more; drinking was defined as the continuous drinking at least once a week for six months or more, while the frequency of physical exercises was defined as the average instances of physical exercises per week [35]; (4) Health service accessibility included the medical service institution closest to home, time to the nearest medical service institution, and whether the participant had contacted a family doctor. The specific questions in the questionnaire can be found in the Additional file 1: Appendix.

### Methodology

#### Lorenz curve and Gini coefficient

The Lorenz curve and Gini coefficient were used to evaluate the equality level of the distribution among populations of persons with varying abilities [36]. Currently, the common methods used to calculate the Gini coefficient include the regional group covariance method and the squared difference method [37]. In view of the non-average grouping of the population and the small number of groups included, this study used the curve fitting method to plot the Lorenz curve and calculated the Gini coefficient of SHOA with IPC using the squared difference method. The calculation formula is:

$$\mathrm G=1-\sum\limits_{\mathrm i=1}^{\mathrm n}P_i{(2Q_{\mathit i}\mathit-W_{\mathit i})}$$

$$Q_i=\sum\limits_{i=1}^nW_i$$

Where "*n*" represents the number of groups; *W*_*i*_ represents the ratio of SHOA score in group *i* to the total score; *P*_*i*_ represents the ratio of the number of respondents in group *i* to the total sample size; *Q*_*i*_ represents the cumulative percentage of *W*_*i*_ from group 1 to group *i*; the sum of *W*_*i*_ and *P*_*i*_ for all groups is 1 (*i* = 1, 2, 3, …, n). The value of the Gini coefficient is between 0 and 1. The smaller the value, the higher the equality of allocation of SHOA with IPC.

#### Vector Autoregressive (VAR) model

VAR model is a method of regressing all current period variables in a model on several lags of all variables and is commonly used to describe the relationship between changes in multivariate time series, researchers have also explored its application to analyze matrix-type cross-sectional data [38, 39]. A p-order VAR model (VAR(p)) is defined as:

$$Y_t=C+{\mathrm\phi}_1Y_{t-1}+{\mathrm\phi}_2Y_{t-2}+\dots{\mathrm\phi}_pY_{t\mathit-p}+{\mathrm\varepsilon}_t,$$

Among them: *Y*_*t*_ is an n × 1-dimensional variable matrix; *C* is an n × 1-dimensional constant vector; and ϕ_i_ is an n × n-dimensional autoregressive coefficient matrix. ε_*t*_ is the n × 1-dimensional random perturbation vector. The intuitive detailed expansion is in the form of:

$$\begin{bmatrix}y_{1t}\\y_{2t}\\\dots\\y_{nt}\end{bmatrix}=\begin{bmatrix}c_1\\c_2\\\dots\\c_n\end{bmatrix}+\begin{bmatrix}\phi_{11}^1&\phi_{12}^1&\dots&\phi_{1n}^1\\\phi_{21}^1&\phi_{22}^1&\dots&\phi_{2n}^1\\\dots&\dots&\dots&\dots\\\phi_{n1}^1&\phi_{n2}^1&\dots&\phi_{nn}^1\end{bmatrix}\begin{bmatrix}y_{1,t-1}\\y_{2,t-2}\\\dots\\y_{n,t-1}\end{bmatrix}+\dots+\begin{bmatrix}\phi_{11}^p&\phi_{12}^p&\dots&\phi_{1n}^p\\\phi_{21}^p&\phi_{22}^p&\dots&\phi_{2n}^p\\\dots&\dots&\dots&\dots\\\phi_{n1}^p&\phi_{n2}^p&\dots&\phi_{nn}^p\end{bmatrix}\begin{bmatrix}y_{1,t-p}\\y_{2,t-p}\\\dots\\y_{n,t-p}\end{bmatrix}+\begin{bmatrix}\varepsilon_{1t}\\\varepsilon_{2t}\\\dots\\\varepsilon_{nt}\end{bmatrix}$$

The application of VAR models requires that the means and variances of the variables used do not vary systematically and periodically [40]. In this study, the Augmented Dickey-Fuller (ADF) test was applied to test the stability of the variables. Also, the Granger causality test was used to test whether there is a correlation between the variables in terms of time series variation.

Based on the results of mean comparisons, LR and Gini coefficient analysis, this study intends to set the age change of old adults as a time series and apply the impulse response function established in the VAR model to analyze whether there is a two-by-two long-term effect between health literacy, health behaviors, health service accessibility, and SHOA with IPC in the process of age change of old adults, and the trend of the long-term effect.

### Analysis tool

The data were entered and processed using the Epidata 3.0 and Excel 2016 software tools. Mean comparison and LR analysis were performed using SPSS 20.0 software. The Lorenz curve, Gini coefficient and VAR method were performed using Excel 2016 and Eviews 6.0 software. *P* < 0.05 was considered a statistically significant difference.

## Results

### Descriptive statistics of the basic situation of old adults

The summary statistics of the basic information of SHOA with IPC were shown in Table 1. 45.62% of the participating older adults were less than or equal to 65 years old, and 51.86% were male. 83.17% of the older adults were married, while 44.23% of the older adults lived mainly in rural areas. The proportions of illiterate older adults and those with at least a college degree were 13.79% and 11.62%, respectively. In terms of pre-retirement occupations and medical insurance, nearly half of seniors were employed in agriculture or unemployed prior to retirement, and more than half of the population purchased basic medical insurance for urban and rural residents. About 50% of the participants reported having used smartphones as their main communication methods, while 7% reported not being used to or never using smartphones. In addition, the analysis found significant differences in SHOA by age, residence, education level, pre-retirement occupation, medical insurance, and communication methods (all *P* < 0.05). The regional analysis revealed non-significant differences in the basic conditions of older adults in Guandong and Guizhou provinces.

**Table 1:** Socio-demographic attributes of the study participants (*N* = 1153)

Index	Total				Hubei	Guangdong	Guizhong
	people (%)	Score^a^ ($\overline x\pm s$)	*t/F*^*e*^	*P*	Score^a^ ($\overline x\pm s$)	Score^a^ ($\overline x\pm s$)	Score^a^ ($\overline x\pm s$)
**Age (years)**							
≤ 65	526 (45.62)	73.74 ± 14.03	3.818	0.022	73.79 ± 14.51**^d^	80.33 ± 9.88	67.29 ± 12.18
65–80	575 (49.87)	75.23 ± 12.61			74.96 ± 12.20	83.15 ± 10.69	69.17 ± 13.32
> 80	52 (3.51)	70.73 ± 10.22			70.28 ± 10.24	82.50 ± 3.54	70.00 ± 10.00
**Sex**							
Male	598 (51.86)	74.45 ± 13.09	0.274	0.784	74.23 ± 13.23	81.32 ± 9.93	69.21 ± 12.30
Female	555 (48.14)	74.24 ± 13.38			74.17 ± 13.15	82.13 ± 10.74	66.59 ± 13.04
**Residence**							
Rural	510 (44.23)	70.90 ± 14.28	-7.925	< 0.001	71.38 ± 14.75***	82.39 ± 9.15	67.90 ± 12.72
Urban	643 (55.77)	77.08 ± 11.62			76.07 ± 11.68	81.53 ± 10.48	76.00 ± 5.48
**Marital status**							
Unmarried	18 (1.56)	71.94 ± 16.19	0.404	0.750	67.27 ± 16.18	85.00 ± 7.07	60.00 ± 14.14
Married	959 (83.17)	74.48 ± 13.16			74.45 ± 13.07	81.10 ± 10.27	68.18 ± 13.03
Divorced	10 (0.87)	75.90 ± 19.34			73.00 ± 7.34	87.50 ± 3.54	-
Widowed and others	166 (14.40)	73.72 ± 12.90			73.49 ± 12.99	86.00 ± 11.5	68.87 ± 10.33
**Education level**							
Illiteracy	159 (13.79)	70.50 ± 14.38	12.843	< 0.001	71.25 ± 14.47***	84.17 ± 10.21*	67.53 ± 13.71
Primary school	344 (29.84)	72.22 ± 14.25			73.04 ± 14.31	74.17 ± 17.69	68.29 ± 12.78
Junior high school	314 (27.23)	74.67 ± 12.58			74.64 ± 12.57	81.25 ± 10.06	67.29 ± 11.61
High school/Technical school	202 (17.52)	77.65 ± 10.71			76.89 ± 11.35	81.59 ± 7.62	73.00 ± 7.15
College degree and above	134 (11.62)	78.64 ± 11.56			75.32 ± 11.77	83.27 ± 9.59	67.53 ± 13.71
**Pre-retirement occupation**^**b**^							
Government staffs	339 (29.40)	77.37 ± 10.99	30.500	< 0.001	75.60 ± 11.02***	81.85 ± 9.76	75.00 ± 5.00
Industry and service workers	245 (21.25)	77.15 ± 12.07			76.97 ± 12.23	79.64 ± 11.54	73.13 ± 8.84
Agricultural staffs and others	569 (49.35)	71.34 ± 14.22			71.98 ± 14.40	83.68 ± 10.78	67.74 ± 12.85
**Medical insurance**							
Basic Medical Insurance for Urban Employees	487 (42.24)	77.72 ± 11.16	30.246	< 0.001	77.33 ± 11.20***	80.23 ± 10.91	77.50 ± 5.00
Basic Medical Insurance for Urban and Rural Residents	640 (55.51)	71.71 ± 14.09			71.29 ± 14.34	82.8 ± 9.16	67.92 ± 12.68
Others	26 (2.25)	76.08 ± 12.38			70.00 ± 9.20	83.17 ± 12.11	68.17 ± 12.62
**Communication methods**^**c**^							
Smartphone	584 (50.65)	77.07 ± 12.68	19.127	< 0.001	75.88 ± 13.06***	81.63 ± 10.10	71.67 ± 12.58
Non-smartphone	498 (43.19)	71.56 ± 13.16			72.50 ± 13.04	81.63 ± 12.47	67.46 ± 12.70
Landline	25 (2.17)	76.00 ± 12.67			74.52 ± 12.44	83.33 ± 15.28	85.00 ± 3.00
Others	46 (3.99)	69.02 ± 13.40			69.75 ± 15.09	-	68.46 ± 12.23

^a^Score: The score of self-rated health of old adults with intact physical condition (0–100); ^b^Pre-retirement occupation: Occupations that older people mainly engaged in before retirement; ^c^Communication methods: The most important tool or means of communication used by older adults in their daily life; ^d^***, **, * indicate significant at 0.01, 0.05 and 0.1 level, respectively; ^e^For two variable classification categories, the independent variable t-test was applied; for three or more variable classification categories, the one-way ANOVA method was applied

### The SHOA with IPC

Table 2 illustrates the SHOA with different health literacy, health behaviors, and health service accessibility status with IPC. In terms of health literacy, the mean SHOA who perceived themselves as actively readers of information related to their health was relatively the highest at 75.92 ± 13.03, while the mean SHOA who perceived themselves as unable to communicate with medical staff was relatively the lowest at 70.75 ± 16.33. Significant differences in SHOA were found between participants who differed in their ability to actively read information related to their health and those who reported making lifestyle changes as guided by the information (*F* = 8.887, *P* < 0.001; *F* = 3.657, *P* = 0.026). In terms of health behaviors, the SHOA who exercised physically seven times a week or more was 76.68 ± 12.52, which may be related to both the ability of exercise to promote health and the already better health of older adults who were able to exercise at a high frequency. In contrast, the analysis revealed no statistically significant difference between SHOA with smoking and drinking statuses (*P* > 0.05). In terms of health service accessibility, there were significant differences in SH among old adults, who differed in the type of nearest medical service institution and time to institution (*F* = 13.295, *P* < 0.001; *F* = 9.726, *P* < 0.001).

**Table 2:** The self-rated health of old adults with intact physical condition

Index		Old adults (people (%))	Score ($\overline x\pm s$)	*t/F*	*P*
**Health literacy**	**Ability to communicate fluently with medical staff**				
	Yes	1015 (88.03)	74.60 ± 13.19	1.916	0.148
	General	106 (9.19)	73.01 ± 12.32		
	No	32 (2.78)	70.75 ± 16.33		
	**Proactive reading of information related to their health**				
	Yes	573 (49.70)	75.92 ± 13.03	8.887	< 0.001
	General	180 (15.61)	73.72 ± 12.22		
	No	400 (34.69)	72.37 ± 13.66		
	**Changing their lifestyle according to the guidance of health information**				
	Yes	468 (40.59)	75.34 ± 13.53	3.657	0.026
	General	307 (26.63)	74.62 ± 11.93		
	No	378 (32.78)	72.90 ± 13.73		
**Health behaviors**	**Smoking status**				
	Smoking	243 (21.08)	73.85 ± 12.93	0.562	0.570
	Quit smoking	66 (5.72)	73.18 ± 13.52		
	No smoking	844 (73.20)	74.58 ± 13.29		
	**Drinking status**				
	Drinking	261 (22.64)	73.64 ± 13.09	2.560	0.078
	Quit drinking	54 (4.68)	71.00 ± 15.52		
	No drinking	838 (72.68)	74.78 ± 13.08		
	**Frequency of physical exercise (Time/Week)**				
	Less than 3	516 (44.75)	71.74 ± 13.53	19.339	< 0.001
	3–6	85 (7.37)	75.05 ± 13.04		
	7 and above	552 (47.88)	76.68 ± 12.52		
**Health services accessible**	**Medical service institution closest to home**				
	Community health service station/village clinic/outpatient department	533 (46.23)	71.50 ± 14.22	13.295	< 0.001
	Community health service center /township health center	158 (13.70)	78.79 ± 11.65		
	County-level and above public medical and health institutions	100 (8.67)	75.44 ± 12.49		
	Pharmacy	339 (29.41)	76.30 ± 11.35		
	Private hospitals, private clinics, et al	23 (1.99)	76.09 ± 14.38		
	**Time to the nearest medical service institution (Minutes)**				
	0–5	181 (15.70)	74.48 ± 13.46	9.726	< 0.001
	5–10	594 (51.52)	75.96 ± 12.73		
	10–15	228 (19.77)	73.67 ± 13.15		
	15–20	53 (4.60)	71.13 ± 12.23		
	> 20	97 (8.41)	67.61 ± 14.06		
	**Whether the participant had contracted a family doctor**				
	Yes	290 (25.15)	73.06 ± 13.67	-1.926	0.054
	No	863 (74.85)	74.78 ± 13.04		
**Total**	-	1153	74.37 ± 13.22	-	-

### Analysis of factors influencing SHOA with IPC

The LR method was used to analyze the personal characteristics level factors influencing SHOA with IPC. Based on the univariate analysis of SHOA and the results of existing studies [13, 14], education level, residence, pre-retirement occupation, medical insurance, and communication methods were included in the regression model as independent variables. In this study, SHOA with IPC was divided into two categories based on the evaluation results, and each SHOA with IPC was assigned a score of 0 or 1. The detailed variable score assignment was shown in Table 3 [13, 14].

**Table 3:** Variables assignment

Variables	Assignment
Self-rated health status	< 85 = 0, ≥ 85 = 1
Age	≤ 65 = 0, 65–80 = 1, > 80 = 2
Residence	Rural = 0, Urban = 1,
Education level	Illiteracy = 1, Primary school = 2, Junior high school = 3, High school/Technical school = 4, College degree and above = 5
Pre-retirement occupation^a^	Government staffs = 1, Industry and service workers = 2, Agricultural staffs and others = 3
Medical insurance	Basic Medical Insurance for Urban Employees = 1, Basic Medical Insurance for Urban and Rural Residents = 2, Others = 3
Communication methods^b^	Smartphone = 1, Non-smartphone = 2, Landline = 3, Others = 4

^a^Pre-retirement occupation: Occupations that older people mainly engaged in before retirement

^b^Communication methods: The most important tool or means of communication used by older adults in their daily life

Regression analysis showed that SHOA with IPC was mainly influenced by age and communication methods (*P* < 0.05). The SH of older adults aged 65 years and younger and 65–80 years was 7.042 and 6.942 times higher than that of older adults aged > 80 years, respectively (*OR* = 7.024, *95%CI* = 1.635–30.340; *OR* = 6.942, *95%CI* = 1.635–29.502). The SHOA whose primary mode of daily communication was the smartphone, was 15.297 times higher than that of older adults whose primary mode of communication was verbal or other (*OR* = 15.297, *95%CI* = 2.035–114.999). Residence, education level, pre-retirement occupation, and medical insurance did not have a significant effect on SHOA with IPC (*P* > 0.05). See Table 4.

**Table 4:** Logistic regression (LR) model for factors associated with SHOA^a^

Indicators	*β*	*SE*	*Wals*	*P*	*OR (95%CI)*
**Age** (> 80 years)	-	-	6.952	0.031	-
≤ 65	1.952	0.745	6.862	0.009	7.042(1.635 ~ 30.340)
65–80	1.938	0.738	6.888	0.009	6.942(1.633 ~ 29.502)
**Residence** (Urban)	0.316	0.240	1.732	0.188	1.372(0.857 ~ 2.198)
**Education level** (College degree and above)	-	-	3.017	0.555	-
Illiteracy	-0.302	0.36	0.702	0.402	0.739(0.365 ~ 1.499)
Primary school	-0.296	0.294	1.014	0.314	0.744(0.418 ~ 1.323)
Junior high school	-0.425	0.266	2.555	0.110	0.654(0.388 ~ 1.101)
High school/Technical school	-0.360	0.253	2.033	0.154	0.697(0.425 ~ 1.145)
**Pre-retirement occupation**^**b**^ (Agricultural staffs and others)	-	-	4.287	0.117	-
Government staffs	-0.212	0.244	0.750	0.387	0.809(0.501 ~ 1.307)
Industry and service workers	0.230	0.223	1.059	0.303	1.258(0.812 ~ 1.950)
**Medical insurance** (Others)	-	-	0.499	0.779	-
Basic Medical Insurance for Urban Employees	-0.169	0.483	0.123	0.726	0.844(0.328 ~ 2.175)
Basic Medical Insurance for Urban and Rural Residents	-0.280	0.486	0.333	0.564	0.756(0.292 ~ 1.957)
**Communication methods**^**c**^ (Others)	-	-	20.817	< 0.001	-
Smartphone	2.728	1.029	7.023	0.008	15.297(2.035 ~ 114.999)
Non-smartphone	1.985	1.023	3.764	0.052	7.281(0.980 ~ 54.094)
Landline	2.440	1.139	4.589	0.032	11.469(1.231 ~ 106.882)
**Constant**	-5.455	1.398	15.223	< 0.001	0.004

^a^SHOA: Self-rated health of old adults

^b^Pre-retirement occupation: Occupations that older people mainly engaged in before retirement

^c^Communication methods: The most important tool or means of communication used by older adults in their daily life

### The equality level for SHOA with IPC’s allocation

**Fig. 1:**
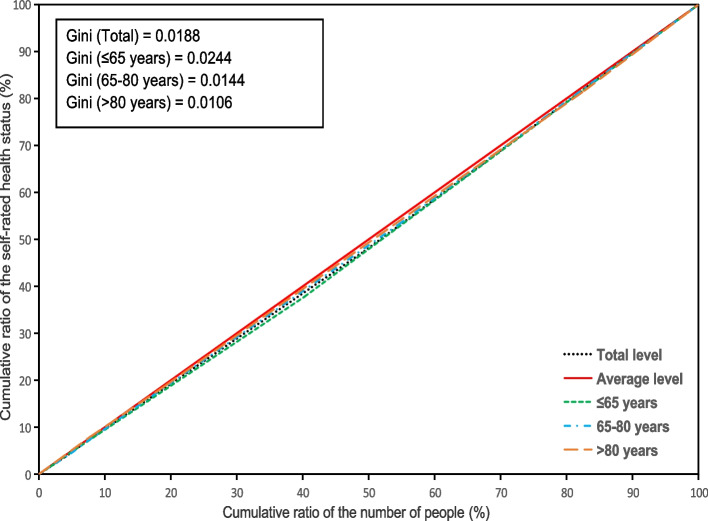
Lorenz curves and Gini coefficients of allocation of self-rated health among older adults with intact physical condition

Based on the results of the above influencing factors analysis, old adults were ranked according to their communication methods, and the specific ranking methods were: others, landline, non-smartphone, and smartphone. The Lorenz curve and Gini coefficients of the allocation of SHOA with IPC based on communication methods are shown in Fig. 1. The total and age-specific Lorenz curves of SHOA with IPC were below the mean curve while the total Gini coefficient for allocation of SHOA with IPC was 0.0188. The analysis by age group showed that the Gini coefficient for allocation of SH based on communication methods was 0.0244, 0.0144, and 0.0106 among older adults aged 60–65, 65–80, and > 80 years, respectively.

### Trend analysis of role based on age change

**Fig. 2:**
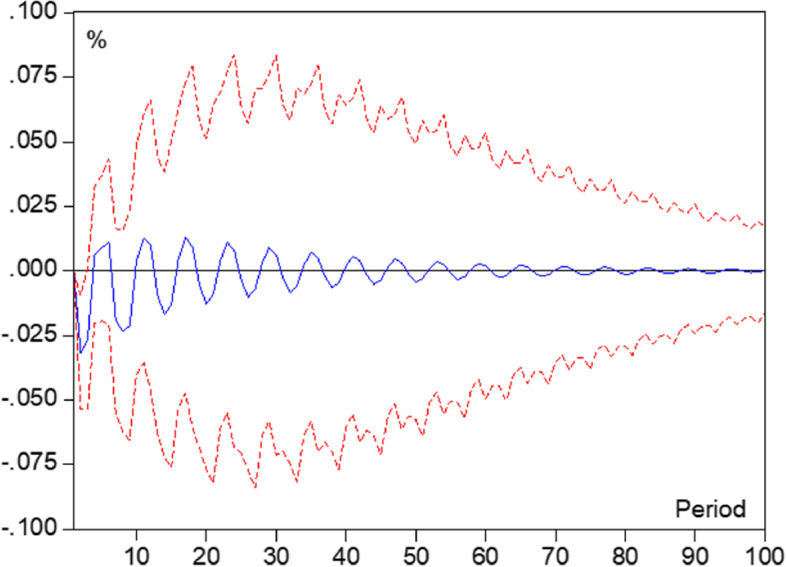
Impulse response of health literacy to self-rated health of old adults

**Fig. 3:**
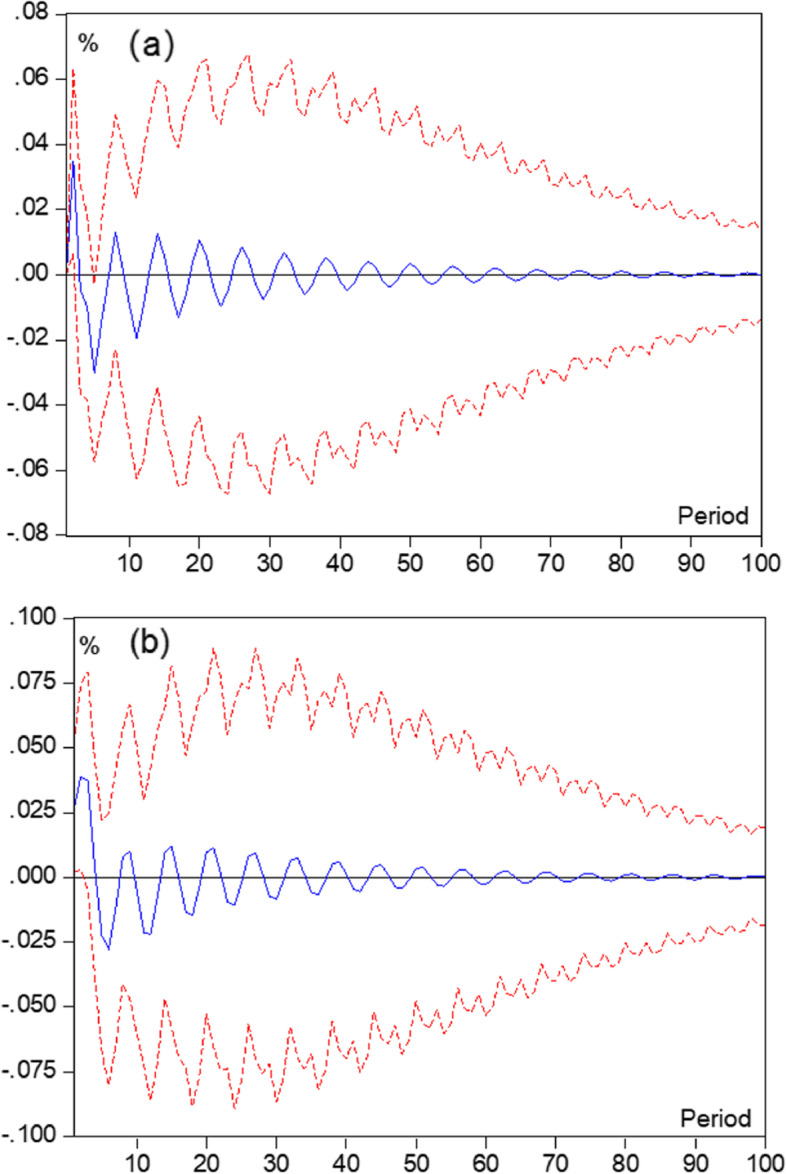
Impulse response of health services accessible to health literacy and health literacy to health behavior. **a** Health services accessible to health literacy; **b** Health literacy to health behavior

The results of existing studies and previous analyses suggest that age, health literacy, health behaviors, and health service accessibility were related to SHOA with IPC, but the trend of causality-based interactions among the factors is not clear. This study developed a VAR model with four variables: health literacy, health behaviors, health service accessibility, and SHOA, using age change as the time series, and analyzed the impulse response function between the factors based on Grant causality. The results of the test for stability of variables applying the Augmented Dickey-Fuller (ADF) method and the ROOT method were shown in the Additional file 1: Appendix File. The results of the analysis based on the Grant causality test showed that there was Grant causality between SHOA and health literacy, health literacy and health behaviors and health service accessibility. The impulse response function showed that the total effect of the change in SH on the health literacy of older adults was negative and the duration of the effect was relatively long (Fig. 2). On the other hand, this study also found the same trend between health literacy and health behaviors and health service accessibility in terms of duration of effect, but the opposite trend in terms of direction of effect (Fig. 3).

## Discussion

### Main findings

The purpose of this study was to assess the current state and equality level of SHOA with IPC and to examine factors associated with them through analysis of data from the “Chinese residents' health service needs survey in the New Era”. The results of the study showed that the state of SH was higher among IPC older adults, but was mainly related to age and communication methods (*P* < 0.05). The impulse response function showed a negative overall effect of change in SH on health literacy in older adults, and the duration of the effect exceeded 50.

Specifically, the total SHOA with IPC in east, central and west China was 74.37 ± 13.22, which is consistent with previous studies [18]. Old adults tend to evaluate their health more by their subjective feelings rather than by corresponding to health criteria item by item, and they have higher SH as long as they do not feel weak in general. On the other hand, they tend to choose reference objects, such as friends, relatives, neighbors, and other older adults in the same age group, to maintain a more positive SH by adjusting the reference objects [41]. The SHOA with IPC was also related to their health literacy, health behaviors, and health service accessibility. The mean SHOA with IPC was found to be highest among those whose nearest medical service institution was a community health service center/township (78.79 ± 11.65), consistent with the lowest SHOA with IPC among those whose time to the nearest medical service institution was greater than 20 min (67.61 ± 14.06). This may be related to the fact that timely and reliable supply of primary/basic health services can make older adults more aware of changes in their health status and reduce their psychological anxiety or self-doubt. Also, older adults who were able to actively read information related to their health and the ability to make lifestyle changes based on information guidance also had relatively high levels of SH [42].

Secondly, age and communication techniques were found to have a substantial impact on SHOA with IPC. The SH of older adults aged 65 years and younger and 65–80 years was 7.042 and 6.942 times higher than that of older adults aged > 80 years, respectively. A similar result was shown in a study by Giron P [43]. Although the decreasing level of health expectations allows older adults to adapt better to the aging process and maintain better SH [15], the accelerated decay of general health status after age > 80 years has led to an abrupt decrease in SH based on the non-linear relationship between physical aging and age [44]. The current study also found that older adults whose primary communication method was smartphones had much higher SH than those whose primary mode of communication was verbal or others. One possible explanation for this is that smartphones provide older adults with easier communication, more health knowledge, and richer entertainment. Based on data derived from CLASS conducted in 2018, a study was undertaken to examine the psycho-social, physical and psychological effects of smartphone usage among old individuals. The findings reveal that smartphone usage was associated with significant reduction in psycho-social loss by 10.10%, and an increase in physical change by 11.70% and psychological gain by 10.40%, compared to non-smartphone users. Furthermore, the findings show that, smartphone use can facilitate the expansion and consolidation of social networks among older adults, thereby promoting favorable perceptions of the aging process [45].

In addition, the allocation of SHOA with IPC based on communication methods was generally equal but tended to favor those who used cell phones, and this trend was relatively more pronounced among the 60–65 population. In China, cell phone usage has already attained widespread adoption. However, a significant disparity exists in smartphone usage among various demographic groups. Specifically, individuals who possess elevated economic status, younger age, and advanced educational attainment are more likely to utilize smartphones. Considering that advanced age is an intrinsic attribute that cannot be modified, policymakers and program planners may prioritize initiatives that enhance the willingness and capacity of older adults, particularly those who are relatively younger, to adopt digital communication technologies such as smartphones.

Finally, the results of the causality-based analysis revealed that the total effect of changes in SH on health literacy among older individuals was unfavorable and lasted for an extended period of time. This may be related to the fact that the improvement of SH can reduce the psychological stress of older adults and thus relax the demands on self [46]. On the other hand, this study also found a causal relationship between health literacy and health behaviors and health service accessibility. This phenomenon could potentially be attributed to the heightened availability of health resources, leading to increased health consciousness among the residents. This, in turn, may generate a subtle, non-coercive reinforcing effect, encouraging the adoption of health-promoting behavioral habits and lifestyles. As such, the authors propose that the government could allocate greater and consistent focus towards individuals exhibiting improved health status, when formulating healthcare policies and delivering services catering to the aging population.

### Strengths and limitations

This study has several strengths and limitations that should be acknowledged. Firstly, the study was specifically tailored towards SHOA with IPC, allowing for a more precise examination of this particular facet of healthcare. Secondly, the utilization of the Gini index as a measure of SHOA allocation equality is a robust and widely recognized approach in healthcare research. Thirdly, the employment of the VAR model for analysis provides a reliable and evidence-based framework for understanding and enhancing the provision of SHOA with IPC. It is important to acknowledge some limitations of this study. Firstly, the stringent data requirement of the VAR methodology utilized, which may restrict its generalizability to other similar settings. Secondly, while the VAR model accounted for various confounding factors, it was unable to fully eliminate the impact of individual variability. Thirdly, SHOA was influenced by many factors at the same time, and this study did not further analyze the potential self-selection or confounding bias that may be existing in the observed data. Fourthly, given the cross-sectional nature of the current study, which precludes the ability to establish causality or evaluate long-term changes over time, the authors suggest that future investigations should further explore and analyses additional crucial factors such as socioeconomic development and advancements in medical technology to enhance the understanding of the topic.

## Conclusions

This study analyzed the current state and influencing factors of SHOA with IPC in east, central and west China, and explored the equality and changing trends of SHOA with IPC based on their influencing factors. SHOA with IPC was shown to be better and was primarily influenced by age and communication methods. Meanwhile, the observed effect of SHOA on health literacy was negative and long-term. To improve SHOA with IPC even further, policymakers could consider promoting the use of modern and convenient communication methods through training and purchasing subsidies. On the other hand, it is also necessary to focus on increasing sustained attention and promoting health literacy and behavior among older adults with improved SH.

## Supplementary Information

**Additional file 1.**

## Data Availability

Availability of data supporting the findings of this study is limited and therefore not publicly available. Data are however available from the corresponding author upon reasonable request.

## Abbreviations

SHOA: Self-rated health of old adults
SH: Self-rated health
IPC: Intact physical condition
LR: Logistic regression
VAR: Vector Autoregression
