# Supplementary material for: Current state, equality level and trends of self-rated health among old adults with intact physical condition
Source: BMC Public Health. 2023 Jun 2;23:1056. doi: 10.1186/s12889-023-15970-8 (PMC10236707; doi:10.1186/s12889-023-15970-8)
Supplement: Supplementary file 1 — Additional file 1. [file 12889_2023_15970_MOESM1_ESM.doc]

**Appendix Table** Unit root test results of variables (0.05)

| Indicators | Inspection standards | *t* | *P* | Critical values | | |
| --- | --- | --- | --- | --- | --- | --- |
|  |  |  |  | 1% level | 5% level | 10% level |
| LN (Self-rated health ) | 2nd difference (D2) | -5.841338 | 0.0001 | -3.752946 | -2.998064 | -2.638752 |
| LN (Health literacy) | 2nd difference (D2) | -7.418276 | <0.0001 | -3.711457 | -2.981038 | -2.629906 |
| LN (Health behavior) | 2nd difference (D2) | -4.717650 | 0.0008 | -3.699871 | -2.976263 | -2.627420 |
| LN (Health services accessible) | 2nd difference (D2) | -5.069103 | 0.0004 | -3.737853 | -2.991878 | -2.635542 |

**Appendix Figure** Test result of ROOT method

**Appendix questionnaire**

**Chinese residents' health service needs survey in the New Era**

**1. Family General Information Questionnaire**

**A1** What is the total number of people living in your household who are usually in the local area? ( )

**A2** How many people are currently living in this household but haven't been registered in the past six months? ( )

**A3** Among the household members, how many of them are working, working or studying outside the county/district and have not lived at home in the past six months? ( )

**A4** What is the nearest medical service provider to your home? (optional)

① Community health service station / village health office / outpatient clinic (health clinic, infirmary)

② Community health service center / township health center

③ County / county city / district health institution

④ Municipal health institution

⑤ provincial and above health institutions

⑥ private hospitals

⑦ private clinics

⑧ Pharmacies

⑨ Other _________________

**A5** How far is the nearest medical service provider to your home?

① Less than 1 km ② 1-2 km ③ 2-3 km ④ 3-4 km ⑤ More than 4 km

**A6** How long (in minutes) does it take to travel from your home to the nearest medical service provider using the most common mode of transportation available? ( )

**A7** What mode of transportation do you and your family members typically use to access the nearest medical service provider?

① Walking

② Using a bicycle or two-wheeled electric vehicle

③ Using a two-wheeled motorcycle or agricultural tricycle

④ Driving a small family car

⑤ Using public transportation

⑥ Other (please specify ________)

**A8** Have you ever signed up with a family doctor?

① Yes ② No

**A9** What is the status of your family land?

① Own land ② Have land, partially rented out

③ Have land, partially rented in ④ All rented out

⑤ All rented out ⑥ None

⑦ Other (non-reimbursable loan, etc.)

**A10** What is your household income for 2017 (urban disposable income, rural net income)? ( )

**2. Personal Information Questionnaire**

**B1** Your gender:

① male ② female

**B2** Your birth year and month (fill in the format: Year. Month YYYY.MM): ( )

**B3** Marital status:

① unmarried ② married ③ divorced ④ widowed ⑤ other

**B4** Are you a local resident? ① Yes ② No

**B5** Nature of your household registration (single choice):

① Agricultural ② Non-agricultural (urban)

③ Now unified as a resident, previously agricultural

④ Non-agricultural (urban) ⑤ No hukou

**B6** Education level:

① No schooling ② Elementary school

③ Junior high school ④ General high school

⑤ Vocational high school/technical school

⑥ Secondary school (secondary school)

⑦ College ⑧ Bachelor

⑨ Graduate and above (master and doctorate)

**B7** Employment status (15 years old and above):

① Working (including flexible employment)

② Retired

③ School students

④ Unemployed or jobless

**B8** Type of occupation (ask for employed and retired persons):

① Organizations, enterprises and institutions in charge

② Professional and technical personnel

③ Clerical and related personnel

④ Commercial/service personnel

⑤ Agriculture, forestry, animal husbandry, fishery and water conservancy production personnel

⑥ Production and transportation equipment operators

⑦ Military

⑧ Other

**B9** Which of the following medical insurance do you participate in? (Multiple choice):

① Basic medical insurance for urban workers

② Basic medical insurance for urban residents

③ New rural cooperative medical insurance

④ Basic medical insurance for urban and rural residents

⑤ Publicly-funded medical insurance

⑥ Commercial medical insurance

⑦ Others (please specify)

⑧ None

**B10** What type of communication tool do you use on daily basis?

① Smartphone ② Non-smartphone ③ Fixed-line phone ④ Other

**3. Health Status**

**C1** How would you describe your current mobility status?

① Walking around without any difficulty

② Some difficulty moving around

③ Can't get out of bed

**C2** How would yoy describe your current ability to perform elf-care activities (such as toileting, dressing, and using the restroom) ?

① No problems ② Some problems ③ Unable to wash or dress yourself

**C3** How would you describe your current ability to perform your usual daily activities (such as work, reading, or housework)?

① No problems ② Some problems ③ Unable to perform daily activities

**C4** How would you rate the level of pain or discomfort you are experiencing in your body currently?

① No pain or discomfort

② Moderate pain or discomfort

③ You feel extremely painful or uncomfortable

**C5** How would you rate your current level of anxiety or depression?

① Do not feel anxious or depressed

② Feel moderately anxious or depressed

③ Feel extremely anxious or depressed

**C6** Please indicate the score on the scale below that best represents your current health status.


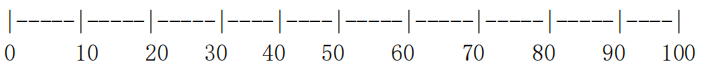


**4. Your health literacy and health behaviors**

**D1** How would you describe the ease of communicating with the medical staff when you receive healthcare services?

① Very not easy ② Not very easy ③ Overly ④ Rather easy ⑤ Very easy

**D2** Do you actively read information related to your own health?

① Not at all ② Not quite ③ Fairly ④ Quite well ⑤ Completely well

**D3** Do you change your lifestyle according to the guidance of health information?

① Not at all ② Not quite ③ Fairly ④ Quite well ⑤ Completely well

**D4** Do you smoke?

① Smoking ② Quit smoking ③ Non-smoking

**D5** Do you drink alcohol ?

① Drinking ② Quit drinking ③ Do not drinking

**D6** On average, how many times per week have you engaged in physical exercise in the past 30 days? ( )
